# Endoscopic management of ureteric stenosis and calculi in bilateral incomplete renal duplication: A case study

**DOI:** 10.37796/2211-8039.1662

**Published:** 2025-09-01

**Authors:** Hafeez Sohaib Ahmad Warraich, Zirwa Younis, Jawairia Warraich, Khizra Warraich

**Affiliations:** a Urology & Renal Transplantation, Allied Hospital Faisalabad Affiliated with Faisalabad Medical University, Faisalabad, Pakistan; b Obstetrics & Gynecology, Allied Hospital Faisalabad Affiliated with Faisalabad Medical University, Faisalabad, Pakistan; c Diagnostic Radiology, Allied Hospital Faisalabad Affiliated with Faisalabad Medical University, Faisalabad, Pakistan; d Internal Medicine, Madinah Teaching Hospital, Faisalabad Affiliated with University Medical and Dental College, Faisalabad, Pakistan

**Keywords:** Bilateral duplex kidneys, Bilateral urolithiasis, Ureteric stenosis, Retrograde intra-renal surgery, Ureteroscopy

## Abstract

Renal duplication anomalies are one of the most frequent congenital urological conditions in the general population all over the world. Bilateral incomplete duplication, however, is exceedingly rare. We present a case of a 29-year-old female with bilateral incomplete duplex kidneys complicated by mid-ureteric stenosis on the right side and calculi in the left ureter and upper moiety of the left duplex kidney. Diagnostic imaging, including ultrasound, computed tomography (CT), and intravenous urography (IVU) confirmed these findings. The patient underwent successful bilateral ureteroscopy (URS) and left-sided retrograde intra-renal surgery (RIRS) for management of the above mentioned complications related to the duplex kidneys in this patient. Postoperative recovery was uneventful, and a further right-sided percutaneous nephrolithotomy (PCNL) was planned. This case underscores the importance of comprehensive diagnostic evaluation and individualized therapeutic strategies in managing complex urological anomalies.

**Categories:**

Urology, Radiology.

## 1. Introduction

Renal duplication anomalies are among the most common congenital urological conditions, observed in approximately 1 %–2 % of the general population [1]. These anomalies manifest either unilaterally or bilaterally, with unilateral ureteral duplication being more frequently encountered in clinical practice [1]. Bilateral duplication remains an exceedingly rare anomaly, with some studies indicating that only 0.3 % of patients are identified with bilateral duplex kidneys via excretory urography [2].

Renal duplication is characterized by the fusion of two distinct renal units (upper and lower) which may exhibit either complete or incomplete ureteral duplication. In cases of complete duplication, the two renal moieties drain separately into the urinary bladder through two separate ureteric orifices, whereas incomplete duplication involves the convergence of the two ureters into a single ureter before draining into the urinary bladder [3]. This condition arises during the 4th to 5th weeks of gestation when either two separate ureteric buds emanate from a single Wolffian duct or a single ureteric bud bifurcates prior to penetrating an aggregation of mesenchymal cells destined to develop into functional nephrons, resulting in complete or incomplete duplication respectively [3].

Duplex collecting systems are predominantly asymptomatic and often incidentally detected. However, duplex systems can be associated with complications such as ureteric obstruction, vesicoureteric reflux, urolithiasis, ectopic ureter leading to incontinence, ureterocele, urinary tract infections and consequent renal scarring [1,4]. Diagnostic evaluations for duplex kidneys are typically initiated in the pediatric population, as symptoms, if present, usually emerge before adulthood. Beyond this period, the condition is more frequently discovered incidentally [5]. Various imaging modalities facilitate diagnosis, with renal tract ultrasound being the initial investigation of choice [5]. Additional imaging may be necessary to further delineate the abnormality, including x-ray kidney-ureter-bladder (KUB), nuclear scans, micturating cystourethrograms (MCUGs), computed tomography (CT), and magnetic resonance imaging (MRI) [5]. The increasing use of routine antenatal ultrasound has augmented the detection rates of duplex kidneys [6].

Given the diverse clinical presentations and surgical indications, numerous surgical strategies have been proposed, each tailored to the specific clinical scenario with varying success rates [7]. Surgical interventions range from minimally invasive endoscopic techniques-such as endoscopic puncture of ureterocele and endoscopic/percutaneous interventions for urolithiasis within duplex systems-to laparoscopic and open surgical approaches encompassing both ablative and reconstructive procedures, including nephrectomy/hemi-nephrectomy, ureteric re-implantation, and pyeloplasty etc. Occasionally, multiple surgical interventions are necessary to address the complications associated with the duplicated collecting system.

This case report presents the surgical management of an adult female with bilateral incomplete duplicated collecting systems, who was successfully treated with endoscopic intervention for a right sided mid-ureteric stenosis and calculi in left ureter and upper moiety of the left duplex kidney.

## 2. Case report

**Fig. 1:**
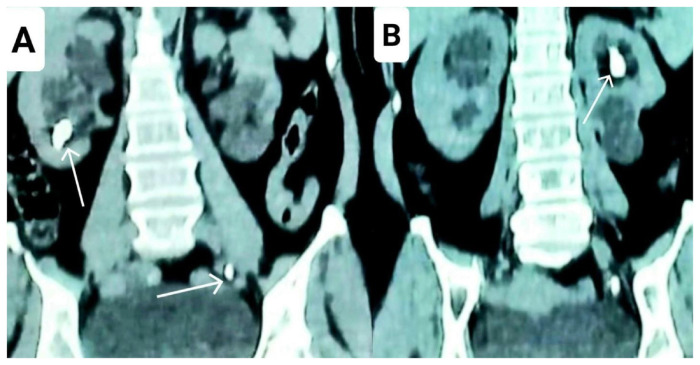
Plain CT kidney-ureter-bladder (KUB) coronal view. A: Calculus measuring 2.2 cm indicated by a white arrow is visible in the lower pole of the lower moiety of the right duplex kidney and another calculus measuring 1 cm indicated by a white arrow is visible in the line of the left ureter at the level of pelvic brim. B: Calculus measuring 1.5 cm indicated by a white arrow is visible in the pelvis of the upper moiety of the left duplex kidney.

A 29-year-old female presented to the urology clinic at Allied Hospital Faisalabad with complaint of bilateral flank pain for the past year. She had been taking self-prescribed oral analgesics with partial relief initially, but recently the pain became refractory to the oral analgesics. A detailed history revealed that the pain was more severe on the left side, and she was recently given injectable analgesics by her local general practitioner (GP). An ultrasound KUB was also advised by her GP, which showed bilateral duplex kidneys with good parenchymal thickness and bilateral moderate hydronephrosis and proximal hydroureter of both the upper and the lower moiety. Right lower moiety had a calculus of 2.2 cm in lower pole, while the renal pelvis of the upper moiety of the left kidney showed a calculus measuring 1.5 cm. Systemic examination and routine blood tests, including serum urea and creatinine, were completely unremarkable. A plain CT KUB was ordered, which confirmed the above-mentioned findings of the ultrasound and also revealed another left ureteric calculus measuring 1 cm at the level of the pelvic brim (Fig. 1A and B). However, it could not be ascertained whether this ureteric calculus was present in the ureter draining the upper or the lower moiety of the left kidney. The Hounsfield units as reported by the radiologist for the right renal, left renal, and left ureteric calculi respectively were 1632 HU, 1486 HU, and 1430 HU.

**Fig. 2:**
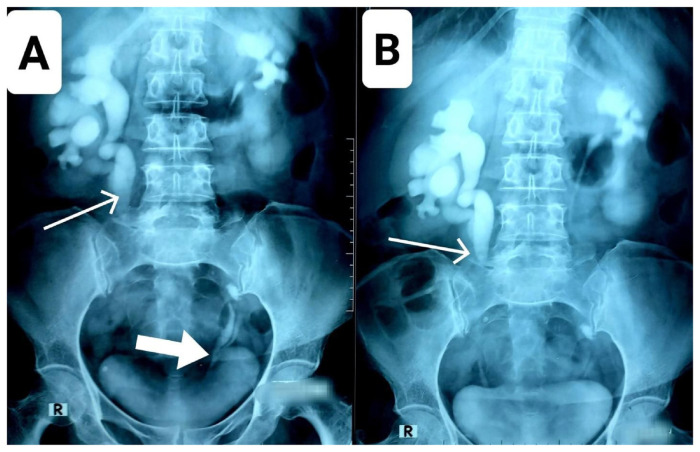
Intravenous urogram (IVU) anteroposterior (AP) view. A: Film taken at 30 min. Each moiety is displaying good uptake of the contrast medium except for the lower moiety of the left duplex kidney. Course of the left sided ureters can be traced down till their union to form a single ureter before entering the urinary bladder, as indicated by a bold white arrow. The contrast delineating the right sided ureters abruptly disappears beyond a point where ureters draining the upper and lower moiety are joining together to form a single ureter suggesting right ureteric stricture/stenosis at this point as is indicated by a thin white arrow. B: Film taken at 60 min, confirms the findings mentioned above.

**Fig. 3:**
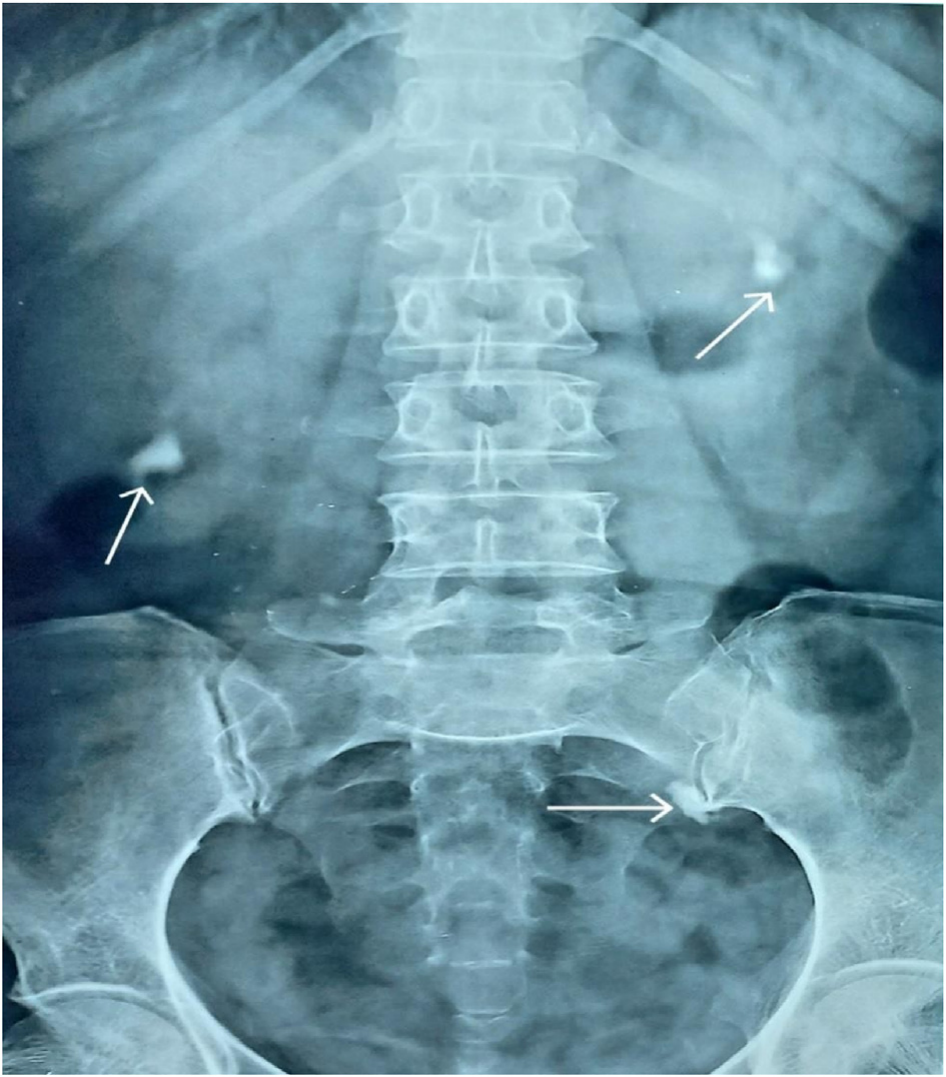
Preoperative plain kidney-ureter-bladder (KUB) x-ray anteroposterior (AP) view. Bilateral renal calculi and a left ureteric calculus are visible as indicated by three white arrows.

Scintigraphy was not performed due to its temporary unavailability at the hospital, but an intravenous urogram (IVU) was ordered, which showed good delineation of each moiety with the contrast medium except for the lower moiety of the left kidney, which showed markedly reduced uptake of the contrast medium (Fig. 2A and B). Also, the duplicated ureters on each side appeared to converge together, implying that duplication of the ureters was incomplete on both sides. The course of the ureters draining the upper and lower moieties of the left duplex kidney was fairly outlined until the left vesicoureteric junction (VUJ), but there was no such outlining on the right side post the point of convergence (at the level of L5) of the ureters draining the upper and lower moieties of the right kidney. This finding on the IVU, along with the right-sided moderate hydronephrosis and proximal hydroureter of both the upper and lower moieties in the absence of any visible left ureteric calculus on plain CT, was convincing enough to suspect ureteric stenosis/stricture at the point of convergence of the right-sided duplicated ureters (see Fig. 3).

A plain KUB x-ray also confirmed the presence of the above-mentioned bilateral renal and left ureteric calculi (Fig. 2), but it was mainly for record-keeping purposes to compare the postoperative radiographs for confirmation of the calculi clearance.

**Fig. 4:**
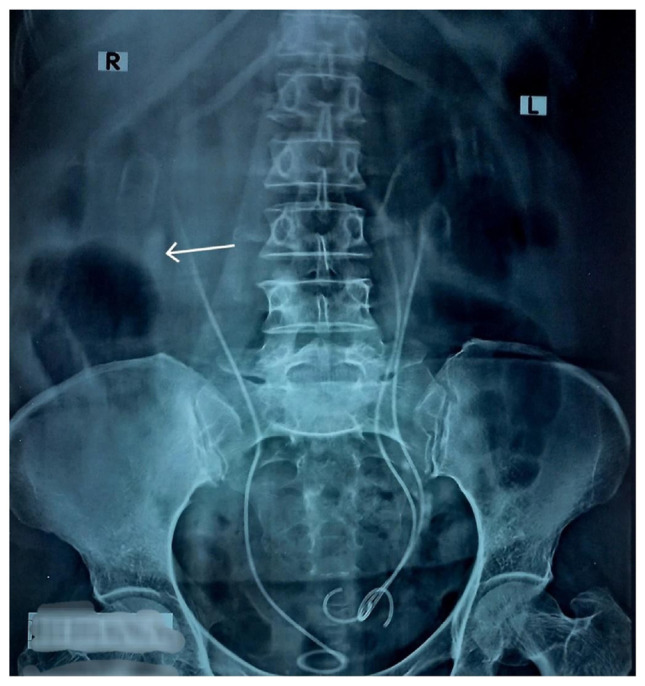
Postoperative plain x-ray kidney-ureter-bladder (KUB) anteroposterior (AP) view. This postoperative plain radiograph is confirming the correct positioning of all three DJ stents, complete clearance of left ureteric and renal calculi and an intact right renal calculus as indicated by a white arrow.

After admission to the urology ward, the patient underwent bilateral ureteroscopy (URS) and left sided retrograde intra-renal surgery (RIRS) using a semi-rigid ureteroscope. URS revealed right-sided midureteric stenosis with a pinpoint opening confirming the provisional diagnosis of right ureteric stenosis/stricture, which was suspected after noting the findings on IVU. Fortunately, it allowed the passage of a hydrophilic guide wire, and subsequently, the stenosis was dilated using an 8Fr polyethylene ureteral dilator. This made the further advancement of the URS possible, confirming that both the ureters draining the upper and lower moieties of the right-sided duplex kidney were joining together to form a single ureter and were otherwise unremarkable except for significant luminal widening likely due to the obstruction caused by the stenosed segment. A double J (DJ) stent was passed up the stenosed segment to allow for its further dilation passively while the right lower moiety calculus owing to its large size was left as such, to be dealt in a 2nd stage surgery i.e. percutaneous nephrolithotomy (PCNL). URS on the left side revealed both the ureters draining the upper and lower moieties of the left duplex kidney were joining together about 4 cm proximal to the left VUJ to form a single ureter. The ureter draining the lower moiety of the left duplex kidney had a partially obstructing calculus of about 1 cm about 6–8 cm proximal to the left VUJ, which was fragmented using a pneumatic lithotripter probe within the semi-rigid ureteroscope, and a DJ stent was passed to prevent the formation of steinstrasse and subsequent obstruction. The ureter draining the upper moiety of the left duplex kidney appeared unremarkable however, the renal pelvis of the upper moiety of the left kidney had a calculus measuring 1.5 cm for which RIRS was done to fragment the stone using the same pneumatic lithotripter within the semi-rigid ureteroscope and DJ stent was passed. Few small fragments of both the ureteric and renal calculus were specially retrieved using an endo-forceps to send them for chemical analysis. A Foley catheter was inserted postoperatively, which was removed on the first postoperative day. A postoperative plain radiograph on the 2nd postoperative day confirmed the correct positioning of all three DJ stents and complete clearance of the left sided ureteric and renal calculus (Fig. 4). The right renal calculus was clearly present at its preoperative position, for which a right-sided PCNL was planned after an interval of 2–3 weeks.

The postoperative period remained uneventful, and the patient was discharged on the third postoperative day with advice to follow up with the urology clinic for discussing the pending results of chemical analysis of the collected stone fragments, removing the DJ stents and readmission for the right sided PCNL.

## 3. Discussion

The initiation of kidney development is marked by the formation of the Wolffian duct, also known as the nephric duct (ND), at the rostral pole of the intermediate mesoderm. During normal development, signaling from the metanephric mesenchyme within the nephrogenic cord induces the formation and outgrowth of a single ureteric bud (UB). The signals emitted from the ureteric bud subsequently prompt the metanephric mesenchyme to differentiate into nephrons, the functional units of the kidney [8]. In cases of incomplete duplication, the two poles of a duplex kidney share the same ureteral orifice in the bladder. These duplex kidneys, featuring a bifid pelvis or ureter, arise when an initially single ureteric bud bifurcates before reaching a population of slightly condensed aggregation of mesenchymal cells within the nephrogenic cord [8]. Conversely, complete duplication means two distinct poles of the kidney draining through two entirely separate ureters that open individually into the urinary bladder. Complete duplication occurs when two separate ureteric buds emerge from a single Wolffian duct [8].

Although renal duplication is among the most prevalent renal anomalies, it remains underreported due to the asymptomatic nature of most cases throughout the lives of the patients [9]. Literature has suggested a possible dominant inheritance pattern of duplex kidney variants, with a higher incidence in females [9]. Symptomatic children may develop severe complications in childhood or early adolescence, including recurrent urinary tract infections that lead to progressive renal function deterioration [9]. In adults, the condition may present with or have the potential to cause future complications such as collecting system obstruction, urolithiasis, ureterocele, ectopic ureter, and vesicoureteral reflux [10]. These complications can lead to a gradual decline in renal function, underscoring the importance of early detection for improved management and survival rates [10]. Knowledge of anatomical variations in duplicated collecting systems is crucial for surgeons and urologists operating on any ureteral pathology. Gynecologists must also be aware of such variations to avoid accidental traumatic injury to the ureter during procedures like hysterectomy. Radiologists need to be familiar with all ureteral variations to accurately interpret radiographs.

Historically, the diagnosis of duplicated ureters was based on excretory urography and retrograde ureteropyelography, but these have now been largely supplanted by CT urography (CTU). Ultrasonography, non-contrast CT (NCCT), and MRI also hold value in the evaluation of duplex kidneys [11]. Despite CTU’s superiority in identifying duplex kidneys compared to NCCT, the “faceless kidney”-an NCCT sign of renal duplicity-remains a well-recognized indicator among radiologists for diagnosing duplex kidneys [12]. This sign represents the structure between the upper and lower moieties of the duplex kidney, devoid of discernible vessels or renal sinus. Treatment and surgical management are highly individualized, tailored to the underlying pathologies. The primary goals are to alleviate clinical symptoms, prevent progressive renal damage, and restore functionality to the upper and lower urinary tracts [13]. Depending on the clinical presentation, which may include obstructive or reflux pathologies, a range of minimally invasive endoscopic, laparoscopic, or open surgical approaches can be employed for either extirpative or reconstructive purposes, or a combination of both, with varying success rates reported in literature [13].

The comprehensive management of this patient highlights the critical importance of meticulous diagnostic evaluation and personalized therapeutic strategies in cases of bilateral duplex kidneys complicated by calculi and ureteric stenosis. Utilizing imaging modalities such as ultrasound, CT, and intravenous urography (IVU) allowed for precise localization and characterization of renal and ureteric calculi, as well as the unexpected identification of ureteric stenosis. Although the lower moiety of the left duplex kidney might have lost some function due to long-standing ureteric obstruction, as evidenced by the relatively lower uptake of contrast medium by this moiety on the IVU films, the safety and efficacy of URS and RIRS in clearing calculi from the left-sided duplex kidney and ureter, as well as negotiating the right-sided ureteric stenosis, highlights the significance of minimally invasive procedures in addressing common complications associated with duplex kidneys. The planned subsequent rightsided PCNL demonstrates a commitment to achieving complete stone clearance and restoring normal renal function. This case signifies the necessity of a multifaceted approach to urological care, integrating advanced imaging, endourological techniques, and thorough postoperative follow-up to ensure optimal patient outcomes. In conclusion, this case report of successful management of bilateral incomplete duplex kidneys with associated calculi and ureteric stenosis using minimally invasive techniques highlights the efficacy of URS and RIRS in similar scenarios. A multidisciplinary approach, incorporating advanced imaging and personalized treatment decisions is crucial for optimal outcomes in such rare and complex cases.
